# Effects of 3-methylmethcathinone on conditioned place preference and anxiety-like behavior: Comparison with methamphetamine

**DOI:** 10.3389/fnmol.2022.975820

**Published:** 2022-07-22

**Authors:** Yang Chen, Libo Zhang, Zengbo Ding, Xianwen Wu, Guibin Wang, Jie Shi

**Affiliations:** ^1^National Institute on Drug Dependence and Beijing Key Laboratory of Drug Dependence, Peking University, Beijing, China; ^2^Department of Pharmacology, School of Basic Medical Sciences, Peking University Health Science Center, Beijing, China; ^3^Shenzhen Public Service Platform for Clinical Application of Medical Imaging, Shenzhen Key Laboratory for Drug Addiction and Medication Safety, Department of Ultrasound, Peking University Shenzhen Hospital, Shenzhen, China; ^4^Department of Laboratory Animal Sciences, Peking University Health Sciences Center, Beijing, China; ^5^Institute of Materia Medica, Chinese Academy of Medical Sciences and Peking Union Medical College, Beijing, China; ^6^The State Key Laboratory of Natural and Biomimetic Drugs, Peking University, Beijing, China; ^7^The Key Laboratory for Neuroscience of the Ministry of Education and Health, Peking University, Beijing, China

**Keywords:** 3-methylmethcathinone, conditioned place preference, elevated plus maze, nucleus accumbens, synaptic transmission

## Abstract

3-Methylmethcathinone (3-MMC), a drug belonging to synthetic cathinones family, raised public attention due to its harmful health effects and abuse potential. Although it has similar properties to other cathinone derivatives, the behavioral effects of 3-MMC remain largely unknown. In the present research, we evaluated the rewarding effect of 3-MMC using conditioned place preference (CPP) paradigm and its effect on anxiety-like behavior using elevated plus maze (EPM) and compared with methamphetamine (METH). Then, we performed a whole-brain c-Fos mapping to identify the specific brain regions in response to 3-MMC exposure and explored the changes of synaptic transmission in nucleus accumbens (NAc) using patch-clamp recording after chronic 3-MMC and METH exposure. 3-MMC induced CPP at higher doses of 3 or 10 mg/kg in rats and acute exposure of 3 mg/kg 3-MMC to rats produced anxiolytic-like effect, while anxiety-like behavior was increased after 7 days of injection with 3-MMC. Whole-brain immunostaining revealed increased c-Fos expression in anterior cingulate cortex (ACC), NAc and ventral tegmental area (VTA) after chronic 3-MMC injection compared with saline, which was similar to METH. Especially, 3-MMC induced more neural activation of VTA compared with METH. Finally, we found that amplitude of spontaneous inhibitory postsynaptic currents (sIPSCs) in NAc was decreased after chronic 3-MMC injection, while frequency of sIPSCs and spontaneous excitatory postsynaptic currents (sEPSCs) were not affected. Taken together, our results revealed the addictive potential of 3-MMC and its effect on anxiety-like behavior, which warn the risks of 3-MMC abuse and justify the control of synthetic cathinones. And 3-MMC selectively inhibit inhibitory but not excitatory transmission onto neurons in NAc, which may contribute to its effects.

## Introduction

As a type of novel psychoactive substance (NPS), synthetic cathinones first appeared in early 21st century and have become increasingly popular in smuggling and illicit drug markets (Assi et al., 2017; Pieprzyca et al., 2020). At the end of 2020, synthetic cathinones were the second largest group among over 830 NPSs detected by the European Monitoring Centre for Drugs and Drug Addiction including 3-Methylmethcathinone (EMCDDA, 2021). 3-methylmethcathinone, also known as 3-MMC or metaphedrone, was a designer drug from the synthetic cathinones family (Ferreira et al., 2019) which first appeared in Sweden in 2012 (Bäckberg et al., 2015) and is currently illegal in majority of nations including France, Poland and China (CFDA, 2015; Pieprzyca et al., 2020; EMCDDA, 2021). There is no known or medical use of 3-MMC, and such drug has attracted public attention around the world because of its harmful health effects and risks of abuse (Adamowicz et al., 2016; Jamey et al., 2016; Dias da Silva et al., 2019; Ferreira et al., 2019; Margasinska-Olejak et al., 2019). To date, there is relatively little literature concerning 3-MMC and it is important to explore its effects to provide sufficient scientific evidence to justify NPSs control.

As one of the NPSs, 3-MMC shares some biological effects with 4-methylmethcathinone (4-MMC), 3,4-methylenedioxy-methamphetamine (MDMA), and methamphetamine (METH) including euphoria, excitement, happiness, increased physical energy, alertness and enhanced awareness (Ferreira et al., 2019). Recently, some researches mentioned that 3-MMC had been related with addiction, several intoxications and even fatalities (Marusich et al., 2012; Ferreira et al., 2019; Margasinska-Olejak et al., 2019; Drevin et al., 2021). Pharmacological study showed that 3-MMC was a monoamine transporter substrate and displayed pronounced dopaminergic and serotonergic activity by inhibition of dopamine (DA) and norepinephrine (NE) uptake (Luethi et al., 2018). The brain dopaminergic system, which plays essential roles in reward, learning and memory, and decision making, is one of the most fundamental theoretical frameworks for drug addiction (Volkow et al., 2011). However, the alternations of neurotransmission remained elusive following 3-MMC administration, particularly in nucleus accumbens (NAc), a crucial downstream target of dopaminergic system and also played a key role in drug addiction (Volkow et al., 2011).

The aim of our research was to investigate the behavioral changes after acute and chronic 3-MMC exposure and its related neural mechanisms. Concretely, we assessed the effects of 3-MMC exposure on conditioned place preference (CPP), locomotor activity and the elevated plus maze (EPM), and compared these with METH. We also evaluated the expression of c-Fos, a marker of stimulus-induced neural activation, in various brain regions linked to addiction, as well as the electrophysiological alternations of NAc neurons after repeated 3-MMC injection.

## Materials and methods

### Animals

Adult male Sprague-Dawley (SD) rats (280–300 g) were purchased from Beijing Vital River Laboratory Animal Technology Co., Ltd. The rats were housed in groups of 4 per cage after arrival with appropriate temperature (22 ± 2°C) and humidity (50 ± 5%), as well as freely accessible water and food. The lighting time was controlled, under a 12-h light/dark cycle. All behavioral experiments were performed during the animal’s dark period. Animal care and experimentation were performed in accordance with the National Institutes of Health Guide for the Care and Use of Laboratory Animals and were approved by the Biomedical Ethics Committee for Animal Use and Protection of Peking University (No: LA2019067).

### Drugs

The METH and 3-MMC were provided by Drug Intelligence and Forensic Center of Ministry of Public Security, China. Both drugs were dissolved in 0.9% saline and ready for intraperitoneal injection.

### Conditioned place preference

The conditioned place preference (CPP) procedure was performed using an unbiased, counterbalanced protocol that has been described previously (Liang et al., 2017). The apparatus for CPP conditioning consisted of 10 identical three-chamber polyvinyl chloride (PVC) boxes. The boxes had two larger chambers (27.9 cm length × 21.0 cm width × 20.9 cm height) that differed in their floor texture (bar or grid, respectively) and the houselights on the walls. The two larger chambers were separated by a smaller chamber (12.1 cm length × 21.0 cm width × 20.9 cm height, with a smooth PVC floor). Baseline preference was assessed by placing the rats in the center chamber of the CPP apparatus and allowing them to explore all three chambers freely for 15 min. Rats that showed a strong unconditioned preference for either side chamber (i.e., >500 s) were excluded from the experiments. On the following 1, 3, 5, and 7 days, the rats received saline, 3-MMC (1, 3, or 10 mg/kg, i.p.), or METH (1 mg/kg, i.p.). Based on the dose of 3-MMC at 0.3 mg/kg for pigs (as 2 mg/kg for rats) in a pharmacological study (Shimshoni et al., 2015; Nair and Jacob, 2016) and intraperitoneal injection of 4-methylethcathinone at 1, 3, or 10 mg/kg in another study (Xu et al., 2016), we chose the dose above for CPP training. All rats received saline injection on the 2, 4, 6, and 8 days. After each injection, rats were immediately confined to the drug-paired or saline-paired conditioning chamber for 45 min before being returned to their home cages. On days 9, rats were placed in the central compartment without receiving any injection and were allowed to explore the entire apparatus freely for 15 min. The CPP score was calculated by the time (in seconds) spent in the drug-paired chamber minus the time spent in the saline-paired chamber during the CPP tests.

### Locomotor activity

Locomotor Activity Test was conducted based on previous study (Deng et al., 2017). All rats were habituated to the locomotor chambers (40 cm × 40 cm × 65 cm) for 3 days (120 min/day) before locomotor activity test. In test day, we first recorded locomotor activity for 30 min, then rats were injected with a single of saline, 3-MMC (1, 3, or 10 mg/kg) or METH (1 mg/kg) and continued to record for 120 min. All locomotor activities were recorded and analyzed with an automated video tracking system (DigBehv-LM4; Shanghai Jiliang Software Technology, Shanghai, China). Locomotor activity is expressed as the total distance traveled in centimeters during a predetermined period of time.

### Elevated plus maze

The elevated plus maze (EPM) was utilized to evaluate anxiety-like behavior based on the rats’ natural fear of open, unprotected, and elevated spaces (Pellow et al., 1985). The EPM consisted of four crossed narrow arms elevated 70 cm from the floor, with two open arms and two closed arms (50 cm long and 10 cm wide). 10 min after acute injection (saline, 3-MMC [1, 3 or 10 mg/kg], or METH [1 mg/kg]) or the last injection of chronic drug administration, each rat was placed in the central zone of the EPM with its head facing an open arm and was allowed to freely explore the maze for 5 min. The time spent in each arm and the traces of rats were analyzed with the EthoVision XT (Noldus IT, Netherlands).

### Immunostaining

Brains were fixed with 4% paraformaldehyde (PFA) for at least 24 h and transferred into phosphate-buffered saline (PBS, pH 7.2) containing 10, 20, and 30% sucrose until they sank. Coronal sections of the brain were cut into 20 μm slice at −20°C in the cryostat (Leica, CM3050 S). Slices were washed with three times of PBS for 5 min, blocked with 5% bovine serum albumin (BSA) dissolved in 0.2% Triton X-100 for 1 h at room temperature and incubated with Rabbit anti-c-Fos (1:500, Abcam, ab190289) primary antibodies dissolved in blocking buffer overnight at 4°C, followed by four times of 15-min wash with PBS at room temperature. After that, slices were incubated with Goat anti-Rabbit Secondary Antibody (Alexa Fluor 488, 1:500, Invitrogen, A-11008) dissolved in blocking buffer for 2 h at room temperature followed by four times of 15-min wash with PBS. Lastly, the slices were mounted by DAPI (Abcam, ab285390) and stored at 4°C for analysis.

Fluorescent images were acquired using a fluorescence microscope (Olympus, Tokyo, Japan) with a 20× objective lens and analyzed according to our previous study (Liang et al., 2017), in which at least three sections were selected from each brain region for each rat. The size of sampled areas for cell quantifications of each brain region from each section was 0.39 mm × 0.39 mm. Brightness and contrast adjustments were applied to the whole image. The number of c-Fos-positive cells were identified and counted in IMARIS software (Oxford Instruments, United Kingdom). An investigator blinded to the experimental conditions performed the image analyses.

### Slice electrophysiology

The experiments were performed as previously described (Yu et al., 2022). Whole-cell patch-clamp recordings of NAc neurons were performed 24 h after the last injection of chronic administration with saline, 1 mg/kg METH or 3 mg/kg 3-MMC. The brains were rapidly removed after anesthetization and 250 μm coronal slices were prepared with a vibratome (Leica VT1200S) in ice-cold solution containing (in mM): 80 NaCl, 26 NaHCO_3_, 3.0 KCl, 1.0 NaH_2_PO_4_, 1.3 MgCl_2_, 1.0 CaCl_2_, 20 D-glucose, and 75 sucrose, saturated with 95% O_2_ and 5% CO_2_. The slices were moved to an incubation chamber containing artificial cerebrospinal fluid (ACSF) consisted of the following (in mM): 124 NaCl, 26 NaHCO_3_, 3.0 KCl, 1.0 NaH_2_PO_4_, 1.3 MgCl_2_, 1.5 CaCl_2_, 20 D-glucose, saturated with 95% O_2_ and 5% CO_2_ at 34°C for 30 min and then at room temperature until used for recording.

For sIPSC recording, the pipette solution contained the following (in mm): 120 CsCl, 20 TEA-Cl, 4 ATP-Mg, 0.3 GTP, 0.5 EGTA, 10 HEPES, and 4.0 QX-314 (pH 7.2, 270–280 mOsm with sucrose). For sEPSC recording, the pipette solution contained the following (in mm): 110 Cs methylsulfate, 15 CsCl, 20 TEA-Cl, 4 ATP-Mg, 0.3 GTP, 0.5 EGTA, 10 HEPES, and 4.0 QX-314 (pH 7.2, 270–280 mOsm with sucrose).

All signals were amplified (Multiclamp 700B, Axon Instruments), filtered at 5 kHz and digitized at 20 kHz (National Instruments Board PCI-MIO-16E4, Igor, Wave Metrics). Data were recorded within Axon pClamp 10 (Molecular Devices, CA, United States). Data analysis referred to our previous research (Yu et al., 2022).

### Statistical analysis

Statistical analyses were performed with GraphPad Prism 9. One-way analysis of variance (ANOVA), two-way ANOVA, or paired *t*-test was used to analyze data when applicable. Bonferroni test was used for *post hoc* analysis after ANOVA. Data were presented as mean ± standard errors of the mean (SEM). Significance was defined as *P < 0.05, ^**^P < 0.01, and ^***^P < 0.001.

## Results

### 3-Methylmethcathinone induced conditioned place preference dose-dependently and increased locomotor activity

**FIGURE 1:**
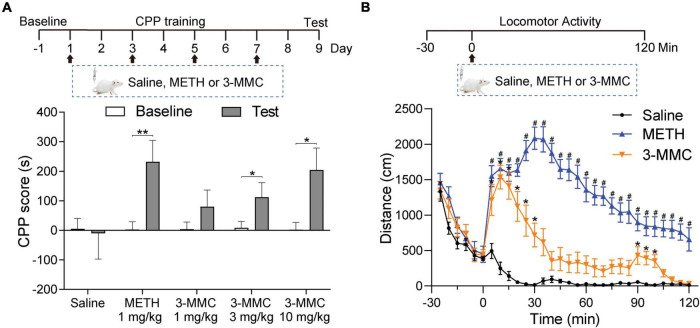
The effects of 3-MMC on CPP and locomotor activity. **(A**) Experimental timeline showing the CPP training. The rats trained with saline, METH (1 mg/kg) or 3-MMC (1, 3, or 10 mg/kg) for 8 days and tested in day 9, paired *t*-test, *P < 0.05, **P < 0.01 significant differences for each drug in CPP test, *n* = 10 for each group. **(B)** A single injection of 3-MMC (3 mg/kg) or METH (1 mg/kg) increased locomotor activity. Each point represents the average distance traveled in 5-min bins. Saline, Meth and 3-MMC group, *post hoc* test, *P < 0.05 significant differences in distance for 3-MMC vs saline, ^#^*P* < 0.05 significant differences for METH vs saline, *n* = 8, 9, and 8, respectively. Data are presented as mean values ± SEM.

Conditioned place preference and locomotor activity are paradigms commonly employed to determine the rewarding and psychomotor properties of psychoactive drugs, respectively (Tzschentke, 2007). To study the rewarding effect of 3-MMC, the rats underwent CPP training (1, 3, 10 mg/kg of 3-MMC; or 1 mg/kg of METH) for 8 days and received CPP test on day 9. Rats with 3 mg/kg 3-MMC, 10 mg/kg 3-MMC and 1 mg/kg METH displayed increased CPP score (paired *t*-test: *t*_9_ = 3.46, ^**^P = 0.0072 for 1 mg/kg METH; *t*_9_ = 2.99, *P = 0.0152 for 3 mg/kg 3-MMC; *t*_9_ = 2.76, *P = 0.0222 for 10 mg/kg 3-MMC), implying that 3-MMC induced CPP dose-dependently (Figure 1A).

Because 3 mg/kg 3-MMC could effectively induce CPP, we next assessed its effect on locomotor activity of rats after acute exposure. A two-way ANOVA of moving distance revealed a main effect of drugs [*F*_(2,22)_ = 97.94, *P* < 0.0001], implying the psychomotor properties of 3-MMC. As shown in Figure 1B, rats treated with 3-MMC exhibited an enhanced locomotor activity from 5 to 30 min and 95–100 min (*post hoc* test: *P < 0.05 for 3-MMC versus saline), while rats with METH injection showed increased activity from 5 to 120 min (*post hoc* test: ^#^*P* < 0.05 for METH versus saline).

### Acute 3-methylmethcathinone injection reduced anxiety-like behavior, while chronic use of 3-methylmethcathinone increased this behavior

**FIGURE 2:**
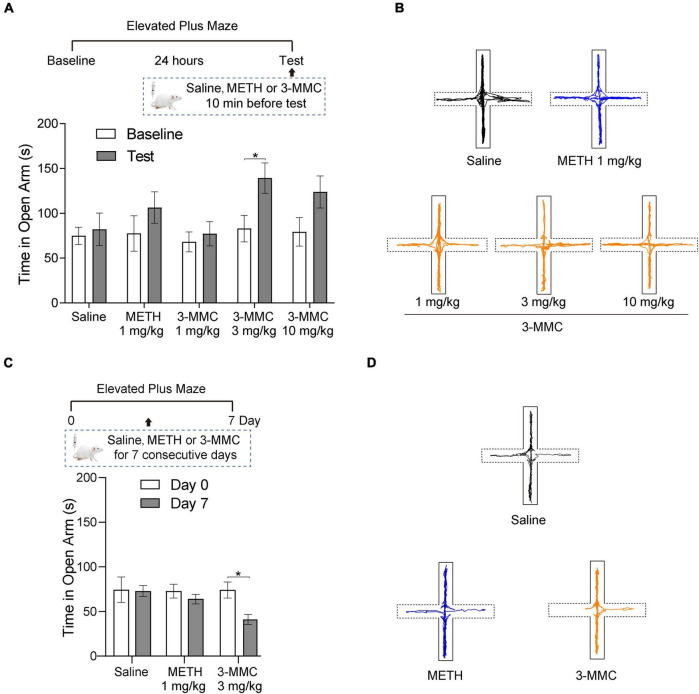
The effects of acute and chronic injection of 3-MMC on EPM. **(A)** A single injection of 3-MMC (3 mg/kg) increased the time in open arms, paired *t*-test, *P < 0.05 significant differences for each drug in EPM test, *n* = 7 for each group. **(B)** Representative diagrams of travel trace in EPM after acute 3-MMC exposure. **(C)** 7 consecutive days of 3-MMC (3 mg/kg) injection decreased the time in open arms, paired *t*-test, *P < 0.05 significant differences for each drug in EPM test, *n* = 9 for each group. **(D)** Representative diagrams of travel trace in EPM after chronic 3-MMC exposure. Data are presented as mean values ± SEM.

To investigate the effect of acute 3-MMC exposure on anxiety-like behavior, we tested the performance of rats in EMP after acute 3-MMC (1, 3, and 10 mg/kg) injection. EMP test showed that rats with 3 mg/kg 3-MMC spent more time in the open arms (paired *t*-test: *t*_6_ = 2.45, *P = 0.0497), implying the rapid anxiolytic-like effect of 3-MMC (Figure 2A). However, the anxiolytic-like effect of 3-MMC instead decreased when the concentration was increased to 10 mg/kg (paired *t*-test: *t*_6_ = 2.00, *P* = 0.092). In addition, there was no change of performance in EMP before and after acute METH exposure (paired *t*-test: *t*_6_ = 1.20, *P* = 0.2763).

We next tested the effect of repeated 3-MMC (3 mg/kg for seven consecutive days) and METH (1 mg/kg for seven consecutive days) exposure on anxiety-like behavior in rats. Result showed that rats administered with chronic 3-MMC spent less time in the open arms (paired *t*-test: *t*_8_ = 3.223, *P = 0.0122), implying that prolonged use of 3-MMC could increase anxiety-like behavior (Figure 2C). And there was no change of performance in EMP before and after chronic METH exposure (paired *t*-test: *t*_8_ = 0.95, *P* = 0.3698). All travel traces of rats in EMP test were presented in Figures 2B,D.

### Region-specific expression of c-Fos after repeated 3-methylmethcathinone injection

Because results above showed that the behavioral changes caused by 3 mg/kg 3-MMC were more significant, we investigated the alternations of neuronal activity after chronic exposure to 3-MMC under this dose. Rats received injection of drugs for 7 days and were sacrificed 90 min after the last injection without any behavioral test. Next, we examined c-Fos expression in brain regions which had been reported to be involved in drug addiction (Piazza and Deroche-Gamonet, 2013; Apaydin et al., 2018) to identify the specific regions in response to chronic 3-MMC exposure.

**FIGURE 3:**
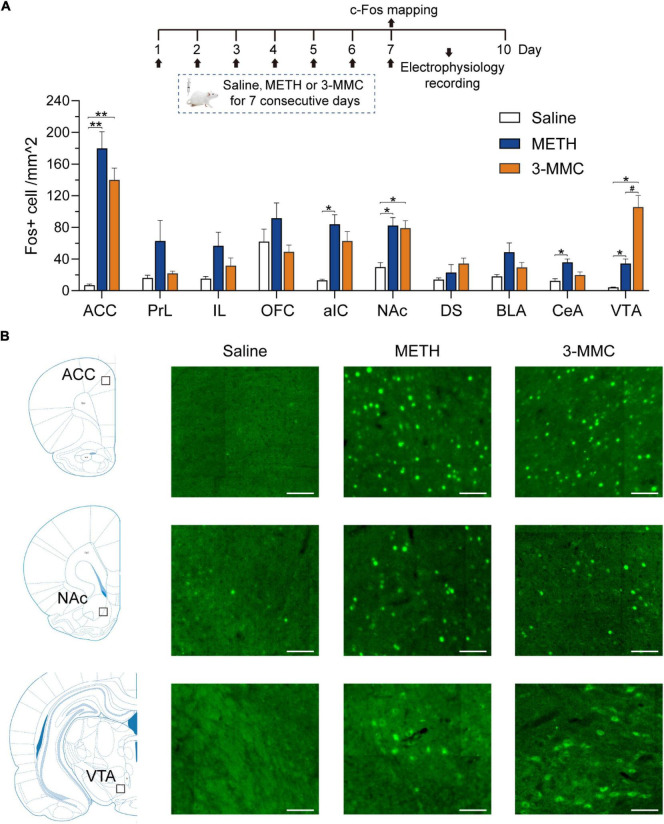
Specific brain regions response to chronic 3-MMC exposure. **(A)** The c-Fos expression of rats 90 min after last injection of drugs, *post hoc* test, *P < 0.05, **P < 0.01 significant differences for 3-MMC vs saline or METH vs saline, ^#^*P* < 0.05 significant differences for 3-MMC vs METH, *n* = 4 for each group. **(B)** Representative immunofluorescent images of c-Fos expression in ACC, NAc, and VTA. Scale bar, 100 μm. ACC, anterior cingulate cortex; PrL, prelimbic cortex; IL, infralimbic cortex; OFC, orbitofrontal cortex; aIC, anterior insular cortex; NAc, nucleus accumbens; DS, dorsal striatum; BLA, basolateral amygdala; CeA, central amygdala; VTA, ventral tegmental area. Data are presented as mean values ± SEM.

As shown in Figures 3A,B, rats with repeated 3-MMC injection presented increased number of c-Fos labeled neurons in anterior cingulate cortex (ACC), NAc, and ventral tegmental area (VTA) compared with saline group [two-way ANOVA revealed a significant interaction effect: *F*_(18,81)_ = 8.35, *P* < 0.0001, *post hoc* test: *P < 0.05 or ^**^P < 0.01 for 3-MMC versus saline group in brain regions above]. Noteworthy, 3-MMC exposure induced more significant activation of VTA than METH (*post hoc* test: ^#^*P* < 0.05 for 3-MMC versus METH), which presented increased expression of c-Fos in ACC, anterior insular cortex (aIC), NAc, central amygdala (CeA) and VTA (*post hoc* test: *P < 0.05 or ^**^P < 0.01 for METH versus saline group in brain regions above). The data of statistics results were shown in Table 1.

**TABLE 1:** The c-Fos expression patterns in each brain region after chronic injection of saline, methamphetamine (METH) and 3-MMC.

Region	c-Fos + cells/mm^2^ (mean ± SEM)			*post hoc* test: *P*		
		
	Saline	METH	3-MMC	Saline VS METH	Saline VS 3-MMC	METH VS 3-MMC
ACC	7.1 ± 1.2	179.7 ± 18.3	139.9 ± 13.0	0.0077**	0.0059**	0.3482
PrL	16.1 ± 3.0	62.6 ± 22.6	21.8 ± 2.6	0.3162	0.4770	0.3850
IL	15.2 ± 2.4	56.6 ± 15.0	31.6 ± 8.5	0.1827	0.3534	0.4768
OFC	61.9 ± 13.8	91.5 ± 16.7	49.0 ± 7.4	0.5063	0.7685	0.2224
aIC	13.0 ± 1.3	83.9 ± 10.6	62.6 ± 10.4	0.0197*	0.0505	0.4758
NAc	29.7 ± 4.9	82.2 ± 8.8	79.0 ± 8.3	0.0167*	0.0169*	0.9707
DS	13.8 ± 2.0	22.9 ± 8.7	34.3 ± 6.0	0.6883	0.1086	0.6423
BLA	18.1 ± 2.1	48.5 ± 10.2	29.4 ± 5.5	0.1534	0.3219	0.4010
CeA	12.3 ± 2.5	35.9 ± 3.7	19.6 ± 3.5	0.0114*	0.3765	0.0712
VTA	4.2 ± 0.3	34.3 ± 5.1	105.6 ± 12.9	0.0285*	0.0130*	0.0251^#^

post hoc test, *P < 0.05, **P < 0.01 significant differences for 3-MMC vs saline or METH vs saline, ^#^P < 0.05 significant differences for 3-MMC vs METH.

### Effects of repeated 3-methylmethcathinone injection on synaptic transmission of nucleus accumbens neurons

**FIGURE 4:**
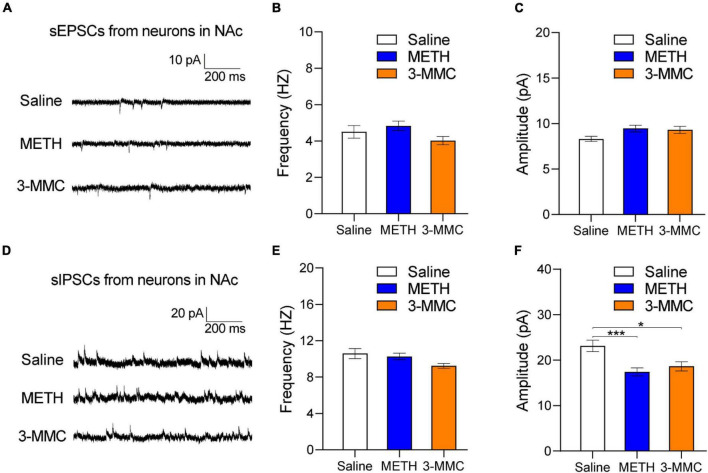
Changes of synaptic transmission in nucleus accumbens (NAc) after chronic 3-MMC injection. **(A)** Representative sEPSCs traces recorded in NAC 24 h after the last injection of drugs. **(B,C)** No difference in the frequency and the amplitude of sEPSCs in NAc after chronic 3-MMC injection. **(D)** Representative sIPSCs traces recorded in NAC 24 h after the last injection of drugs. **(E,F)** The amplitude of sIPSCs was decreased after chronic 3-MMC injection without change of frequency of sIPSCs, *post hoc* test, *P < 0.05, ***P < 0.001 significant differences for 3-MMC vs saline and METH vs saline respectively. For slice electrophysiology: 25 neurons from three rats with saline injection, 32 neurons from three rats with METH injection and 27 neurons from three rats with 3-MMC injection. Data are presented as mean values ± SEM.

As psychoactive substance, both 3-MMC and METH induced activation of NAc neurons after chronic exposure. However, there is no research about the effect of 3-MMC on NAc yet, a key structure in rewarding, motivation, and incentivized learning (Volkow et al., 2011). To determine the effects of 3-MMC on synaptic activity, we recorded spontaneous excitatory and inhibitory postsynaptic currents (sEPSCs and sIPSCs) in NAc. As shown in Figures 4E,F, the amplitude of sIPSCs was decreased after repeated 3-MMC exposure [one-way ANOVA, *F*_(2,81)_ = 7.97, *P* = 0.0007, *post hoc* test: *P < 0.05 for saline versus 3-MMC, ^***^P < 0.001 for saline versus METH, *P* = 0.7895 for 3-MMC vs METH], while frequency of sIPSCs remained unchanged [one-way ANOVA, *F*_(2,81)_ = 3.02; *P* = 0.0541]. Neither the frequency [one-way ANOVA, *F*_(2,81)_ = 2.17; *P* = 0.1207] nor the amplitude of sEPSCs [one-way ANOVA, *F*_(2,81)_ = 2.97; *P* = 0.0569] was affected by repeated 3-MMC exposure (Figures 4B,C). The representative sEPSCs and sIPSCs traces of NAc neurons were presented in Figures 4A,D.

## Discussion

3-Methylmethcathinone is a recently emerged cathinone derivative which is introduced initially to replace 4-MMC, and is legally controlled in many countries, but is still easily available for purchase from websites or entertainment venues (Ferreira et al., 2019). Most 3-MMC consumption concerns abuse, and its related mortality has alarmingly increased in recent years (Ferreira et al., 2019; Margasinska-Olejak et al., 2019; Drevin et al., 2021). Previous information on biological effects of 3-MMC was scarce, and most derived from humans, including case reports of intoxicated individuals that were admitted to the emergency, online questionnaires and self-reports of consumers. Our study evaluated the abuse potential of 3-MMC in rats using representative addictive model of CPP (Tzschentke, 2007) for the first time, and clearly demonstrated that 3-MMC induced CPP in a dose-dependent manner, indicating the rewarding effect of 3-MMC. In addition, rats with acute 3 mg/kg 3-MMC exposure increased locomotor activity, which lasted shorter than METH and reappeared after the first increase. It may be associated with the different effects of their metabolites. Within 30–45 min of METH injection, it reached maximum concentration in brain dialysate (El-Sherbeni et al., 2020) and was metabolized to amphetamine with similar psychoactive property as METH and para-hydroxymethamphetamine (p-OHMA) without psychoactive activity (Shima et al., 2008). Metabolism of METH explained its long-lasting psychoactive effects, which was consistent with the time-locomotor activity curve in present study. Within 5–10 min of 3-MMC oral ingestion, it reached peak concentration in plasma (Shimshoni et al., 2015) displayed its pharmacological characteristics of rapid absorption. Although 3-methylephedrine and 3-methylnorephedrine were identified as mainly metabolites of 3-MMC (Frison et al., 2016), their effects on locomotor activity have not been investigated yet. Further study of these metabolites may provide clear explanation for the effects of 3-MMC.

The desired effects of 3-MMC are euphoria, excitement, improved social skills and feelings of empathy, while chronic abuse may trigger deterioration of relationships with others, tachycardia, agitation, depression, and anxiety (Shimshoni et al., 2015; Sande, 2016; Ferreira et al., 2019; Drevin et al., 2021). We observed acute exposure of 3 mg/kg 3-MMC decreased anxiety-like behavior of rats, which has been reported for other synthetic cathinones such as mephedrone (Pail et al., 2015) and *N*-ethyl-pentedrone (Sande, 2016). This anxiolytic-like effect may be subjectively interpreted as a positive and euphoria experience by the consumers and influence on the further abuse. Interestingly, it seemed that the acute anxiolytic ability of 3-MMC decreased after exposure to 10 mg/kg of 3-MMC, implying the complex interaction between use of synthetic cathinones and adverse psychiatric sequelae. However, chronic administration of 3-MMC increased anxiety-like behavior, which was consistent with the problems users experienced (Sande, 2016).

Whole brain c-Fos mapping of present study showed that ACC, VTA, and NAc were significantly activated after chronic use of 3-MMC, which was consistent with its amphetamine-like stimulant properties (Ferreira et al., 2019; Espinosa-Velasco et al., 2022). Dopamine-releasing neurons of the VTA have central roles in reward-related and goal-directed behaviors (Morales and Margolis, 2017), and a major reward-related output of VTA neurons is NAc (Cooper et al., 2017). Craving and impulsive behavior are related to increased neural activity in ACC (Zhao et al., 2020). Previous studies reported that 3-MMC could induce release of NE, serotonin (5-HT) and DA by inhibition of the reuptake of monoamines *via* DA, NE, and 5-HT transporters (Luethi et al., 2018; Zwartsen et al., 2020), which may be the mechanism of 3-MMC addiction. Considering the enhancing effect on neuronal activity and rewarding of monoamines (Waterhouse and Navarra, 2019; Liu et al., 2020), 3-MMC may produce rewarding effect *via* increasing monoamines to activate ACC, VTA, and NAc, and demonstrated abuse liability. Compared with METH, rats treated with 3-MMC produced more activation of VTA, a region plays a role in both addiction and anxiety (Tovote et al., 2015; Salamone et al., 2016). Previous study reported that chemogenetic activation of VTA dopaminergic neurons directly triggered anxiety-like behavior (Qi et al., 2022), which explained that increased anxiety-like behavior after chronic 3-MMC exposure, implying pronounced effect of 3-MMC on dopaminergic neurons.

Nucleus accumbens plays a crucial role in addiction because it participates in the motivation, incentive salience, positive reinforcement, reward and reinforcement learning (Carlezon and Thomas, 2009; Salamone et al., 2016). Present and previous study found that chronic exposure of both 3-MMC and METH (Miliano et al., 2016) could activate NAc neurons. However, alterations of these neurons after chronic 3-MMC exposure are largely unknown. To the best of our knowledge, this is the first report of functional alterations of NAc neurons after chronic 3-MMC exposure in rats. Our results showed that sIPSCs of NAc neurons were decreased by chronic 3-MMC exposure without a change of sEPSCs, suggesting that 3-MMC inhibited inhibitory but not affecting excitatory neurotransmission. Rats with 7 days of 3-MMC injection exhibited reduced amplitude of sIPSCs, while the frequency of sIPSCs was not changed for NAc neurons, implying decreased postsynaptic neurotransmission by 3-MMC, without affecting presynaptic GABAergic transmitter release at the synapse of NAc (Gantz et al., 2013). Due to the complex neuron types in NAc, the precise neuronal mechanisms of 3-MMC addiction and its relationship with mental disorders need to be further studied.

There were two limitations in our study. One was that we performed the c-Fos mapping after chronic injection of 3-MMC without any behavioral test, which weakened the linking between 3-MMC induced neuronal activity and its effects on behavior of rats. The other was that the higher dose of 3-MMC should be introduced, such as 50 or 150 mg/kg. Because clinical study found that over half of the respondents consumed more than 0.5 g of 3-MMC (as 50 mg/kg for rats) in a single evening and 26.2% of those users reported more than 1.5 g of 3-MMC (as 150 mg/kg for rats) per day (Nair and Jacob, 2016; Sande, 2016).

In summary, our results revealed that 3-MMC has addictive potential due to its rewarding effects, which was related to activation of ACC, NAc and VTA after chronic exposure. Acute administration of 3 mg/kg 3-MMC produced anxiolytic-like effects, while chronic use of 3-MMC increased anxiety-like behavior, which may be related with hyperactivation of VTA. Moreover, the post-synaptic transmission of inhibitory neurons in the NAc might be involved in the mechanisms of chronic use of 3-MMC. Overall, all these findings are warning about the risks of 3-MMC consumption and encourage scientists to carry out further studies to fully elucidate the addictive potential, the neurochemical changes in the whole brain and other effects of this novel synthetic cathinone.

## Data availability statement

The original contributions presented in this study are included in the article/supplementary material, further inquiries can be directed to the corresponding authors.

## Ethics statement

The animal study was reviewed and approved by Biomedical Ethics Committee for Animal Use and Protection of Peking University.

## Author contributions

YC and JS conceived the project. YC and LZ provided experimental design. YC and ZD performed the experiments. YC and XW contributed to analysis of the data. YC and GW wrote the manuscript with input from all authors. All authors contributed to the article and approved the submitted version.

## Conflict of interest

The authors declare that the research was conducted in the absence of any commercial or financial relationships that could be construed as a potential conflict of interest.

## Publisher’s note

All claims expressed in this article are solely those of the authors and do not necessarily represent those of their affiliated organizations, or those of the publisher, the editors and the reviewers. Any product that may be evaluated in this article, or claim that may be made by its manufacturer, is not guaranteed or endorsed by the publisher.

## References

[B1] Adamowicz P. Gieron J. Gil D. Lechowicz W. Skulska A. Tokarczyk B. (2016). 3-Methylmethcathinone–interpretation of blood concentrations based on analysis of 95 cases. *J. Anal. Toxicol.* 40 272–276. 10.1093/jat/bkw018 26989222

[B2] Apaydin N. Ustun S. Kale E. H. Celikag I. Ozguven H. D. Baskak B. (2018). Neural mechanisms underlying time perception and reward anticipation. *Front. Hum. Neurosci.* 12:115. 10.3389/fnhum.2018.00115 29662447 PMC5890198

[B3] Assi S. Gulyamova N. Kneller P. Osselton D. (2017). The effects and toxicity of cathinones from the users’ perspectives: a qualitative study. *Hum. Psychopharmacol.* 32:e2610. 10.1002/hup.2610 28631397

[B4] Bäckberg M. Lindeman E. Beck O. Helander A. (2015). Characteristics of analytically confirmed 3-MMC-related intoxications from the Swedish STRIDA project. *Clin. Toxicol.* 53 46–53. 10.3109/15563650.2014.981823 25422862

[B5] Carlezon W. A. Jr. Thomas M. J. (2009). Biological substrates of reward and aversion: a nucleus accumbens activity hypothesis. *Neuropharmacology* 56 (Suppl. 1), 122–132. 10.1016/j.neuropharm.2008.06.075 18675281 PMC2635333

[B6] CFDA (2015). *Measures for the List of Non-Medicinal Narcotic Drugs and Psychotropic Substances.* Beijing: China Food and Drug Administration.

[B7] Cooper S. Robison A. J. Mazei-Robison M. S. (2017). Reward circuitry in addiction. *Neurotherapeutics* 14 687–697. 10.1007/s13311-017-0525-z 28324454 PMC5509624

[B8] Deng J. H. Yan W. Han Y. Chen C. Meng S. Q. Sun C. Y. (2017). Predictable chronic mild stress during adolescence promotes fear memory extinction in adulthood. *Sci. Rep.* 7:7857. 10.1038/s41598-017-08017-7 28798340 PMC5552791

[B9] Dias da Silva D. Ferreira B. Roque Bravo R. Rebelo R. Duarte de Almeida T. Valente M. J. (2019). The new psychoactive substance 3-methylmethcathinone (3-MMC or metaphedrone) induces oxidative stress, apoptosis, and autophagy in primary rat hepatocytes at human-relevant concentrations. *Arch. Toxicol.* 93 2617–2634. 10.1007/s00204-019-02539-x 31468101

[B10] Drevin G. Rossi L. H. Ferec S. Briet M. Abbara C. (2021). Chemsex/slamsex-related intoxications: a case report involving gamma-hydroxybutyrate (GHB) and 3-methylmethcathinone (3-MMC) and a review of the literature. *Forensic Sci. Int.* 321:110743. 10.1016/j.forsciint.2021.110743 33640780

[B11] El-Sherbeni A. A. Stocco M. R. Wadji F. B. Tyndale R. F. (2020). Addressing the instability issue of dopamine during microdialysis: the determination of dopamine, serotonin, methamphetamine and its metabolites in rat brain. *J. Chromatogr. A* 1627 461403. 10.1016/j.chroma.2020.461403 32823108 PMC7484461

[B12] EMCDDA (2021). *European Drug Report 2021: Trends and Developments.* Lisbon: European Monitoring Centre for Drugs and Drug Addiction.

[B13] Espinosa-Velasco M. Reguilón M. D. Bellot M. Nadal-Gratacós N. Berzosa X. Gómez-Canela C. (2022). Repeated administration of N-ethyl-pentedrone induces increased aggression and impairs social exploration after withdrawal in mice. *Prog. Neuropsychopharmacol. Biol. Psychiatry* 117:110562. 10.1016/j.pnpbp.2022.110562 35500841

[B14] Ferreira B. Dias da Silva D. Carvalho F. de Lourdes Bastos M. Carmo H. (2019). The novel psychoactive substance 3-methylmethcathinone (3-MMC or metaphedrone): a review. *Forensic Sci. Int.* 295 54–63. 10.1016/j.forsciint.2018.11.024 30572220

[B15] Frison G. Frasson S. Zancanaro F. Tedeschi G. Zamengo L. (2016). Detection of 3-methylmethcathinone and its metabolites 3-methylephedrine and 3-methylnorephedrine in pubic hair samples by liquid chromatography-high resolution/high accuracy Orbitrap mass spectrometry. *Forensic Sci. Int.* 265 131–137. 10.1016/j.forsciint.2016.01.039 26901638

[B16] Gantz S. C. Bunzow J. R. Williams J. T. (2013). Spontaneous inhibitory synaptic currents mediated by a G protein-coupled receptor. *Neuron* 78 807–812. 10.1016/j.neuron.2013.04.013 23764286 PMC3697754

[B17] Jamey C. Kintz P. Martrille L. Raul J. S. (2016). Fatal Combination with 3-Methylmethcathinone (3-MMC) and Gamma-Hydroxybutyric Acid (GHB). *J. Anal. Toxicol.* 40 546–552. 10.1093/jat/bkw058 27405362

[B18] Liang J. Li J. L. Han Y. Luo Y. X. Xue Y. X. Zhang Y. (2017). Calpain-GRIP signaling in nucleus accumbens core mediates the reconsolidation of drug reward memory. *J. Neurosci.* 37 8938–8951. 10.1523/jneurosci.0703-17.2017 28821652 PMC6596794

[B19] Liu Z. Lin R. Luo M. (2020). Reward contributions to serotonergic functions. *Annu. Rev. Neurosci.* 43 141–162. 10.1146/annurev-neuro-093019-112252 32640931

[B20] Luethi D. Kolaczynska K. E. Docci L. Krahenbuhl S. Hoener M. C. Liechti M. E. (2018). Pharmacological profile of mephedrone analogs and related new psychoactive substances. *Neuropharmacology* 134(Pt A), 4–12. 10.1016/j.neuropharm.2017.07.026 28755886

[B21] Margasinska-Olejak J. Celinski R. Fischer A. Stojko J. (2019). A fatal case of poisoning of a 19-year-old after taking 3-MMC. *Forensic Sci. Int.* 300 e34–e37. 10.1016/j.forsciint.2019.02.040 31056341

[B22] Marusich J. A. Grant K. R. Blough B. E. Wiley J. L. (2012). Effects of synthetic cathinones contained in “bath salts” on motor behavior and a functional observational battery in mice. *Neurotoxicology* 33 1305–1313. 10.1016/j.neuro.2012.08.003 22922498 PMC3475178

[B23] Miliano C. Serpelloni G. Rimondo C. Mereu M. Marti M. De Luca M. A. (2016). Neuropharmacology of new psychoactive substances (NPS): focus on the rewarding and reinforcing properties of cannabimimetics and amphetamine-like stimulants. *Front. Neurosci.* 10:153. 10.3389/fnins.2016.00153 27147945 PMC4835722

[B24] Morales M. Margolis E. B. (2017). Ventral tegmental area: cellular heterogeneity, connectivity and behaviour. *Nat. Rev. Neurosci.* 18 73–85. 10.1038/nrn.2016.165 28053327

[B25] Nair A. B. Jacob S. (2016). A simple practice guide for dose conversion between animals and human. *J. Basic Clin. Pharm.* 7 27–31. 10.4103/0976-0105.177703 27057123 PMC4804402

[B26] Pail P. B. Costa K. M. Leite C. E. Campos M. M. (2015). Comparative pharmacological evaluation of the cathinone derivatives, mephedrone and methedrone, in mice. *Neurotoxicology* 50 71–80. 10.1016/j.neuro.2015.08.004 26254738

[B27] Pellow S. Chopin P. File S. E. Briley M. (1985). Validation of open:closed arm entries in an elevated plus-maze as a measure of anxiety in the rat. *J. Neurosci. Methods* 14 149–167. 10.1016/0165-027090031-7 2864480

[B28] Piazza P. V. Deroche-Gamonet V. (2013). A multistep general theory of transition to addiction. *Psychopharmacology* 229 387–413. 10.1007/s00213-013-3224-4 23963530 PMC3767888

[B29] Pieprzyca E. Skowronek R. Nižnanský L’. Czekaj P. (2020). Synthetic cathinones - From natural plant stimulant to new drug of abuse. *Eur. J. Pharmacol.* 875:173012. 10.1016/j.ejphar.2020.173012 32087255

[B30] Qi G. Zhang P. Li T. Li M. Zhang Q. He F. (2022). NAc-VTA circuit underlies emotional stress-induced anxiety-like behavior in the three-chamber vicarious social defeat stress mouse model. *Nat. Commun.* 13:577. 10.1038/s41467-022-28190-2 35102141 PMC8804001

[B31] Salamone J. D. Pardo M. Yohn S. E. López-Cruz L. SanMiguel N. Correa M. (2016). Mesolimbic dopamine and the regulation of motivated behavior. *Curr. Top. Behav. Neurosci.* 27 231–257. 10.1007/7854_2015_383 26323245

[B32] Sande M. (2016). Characteristics of the use of 3-MMC and other new psychoactive drugs in Slovenia, and the perceived problems experienced by users. *Int. J. Drug Policy* 27 65–73. 10.1016/j.drugpo.2015.03.005 25908121

[B33] Shima N. Katagi M. Kamata H. Zaitsu K. Kamata T. Nishikawa M. (2008). Urinary excretion of the main metabolites of 3,4-methylenedioxymethamphetamine (MDMA), including the sulfate and glucuronide of 4-hydroxy-3-methoxymethamphetamine (HMMA), in humans and rats. *Xenobiotica* 38 314–324. 10.1080/00498250701802506 18274959

[B34] Shimshoni J. A. Britzi M. Sobol E. Willenz U. Nutt D. Edery N. (2015). 3-Methyl-methcathinone: pharmacokinetic profile evaluation in pigs in relation to pharmacodynamics. *J. Psychopharmacol.* 29 734–743. 10.1177/0269881115576687 25804420

[B35] Tovote P. Fadok J. P. Lüthi A. (2015). Neuronal circuits for fear and anxiety. *Nat. Rev. Neurosci.* 16 317–331. 10.1038/nrn3945 25991441

[B36] Tzschentke T. M. (2007). Measuring reward with the conditioned place preference (CPP) paradigm: update of the last decade. *Addict. Biol.* 12 227–467. 10.1111/j.1369-1600.2007.00070.x 17678505

[B37] Volkow N. D. Wang G. J. Fowler J. S. Tomasi D. Telang F. (2011). Addiction: beyond dopamine reward circuitry. *Proc. Natl. Acad. Sci. U.S.A.* 108 15037–15042. 10.1073/pnas.1010654108 21402948 PMC3174598

[B38] Waterhouse B. D. Navarra R. L. (2019). The locus coeruleus-norepinephrine system and sensory signal processing: a historical review and current perspectives. *Brain Res.* 1709 1–15. 10.1016/j.brainres.2018.08.032 30179606

[B39] Xu P. Qiu Y. Zhang Y. B ai Y. Xu P. Liu Y. (2016). The Effects of 4-methylethcathinone on conditioned place preference, locomotor sensitization, and anxiety-like behavior: a comparison with methamphetamine. *Int. J. Neuropsychopharmacol.* 19:yv120. 10.1093/ijnp/pyv120 26612552 PMC4851266

[B40] Yu Z. Han Y. Hu D. Chen N. Zhang Z. Chen W. (2022). Neurocan regulates vulnerability to stress and the anti-depressant effect of ketamine in adolescent rats. *Mol. Psychiatry* 27 2522–2532. 10.1038/s41380-022-01495-w 35264728

[B41] Zhao Y. Sallie S. N. Cui H. Zeng N. Du J. Yuan T. (2020). Anterior cingulate cortex in addiction: new insights for neuromodulation. *Neuromodulation* 24 187–196. 10.1111/ner.13291 33090660

[B42] Zwartsen A. Olijhoek M. E. Westerink R. H. S. Hondebrink L. (2020). Hazard characterization of synthetic cathinones using viability, monoamine reuptake, and neuronal activity assays. *Front. Neurosci.* 14:9. 10.3389/fnins.2020.00009 32063828 PMC7000521

